# Hospitalisation rates for epilepsy, asthma and insulin-dependent diabetes in 796 190 school-aged children and young people with and without intellectual disabilities: a record-linkage cohort study

**DOI:** 10.1136/bmjopen-2024-088809

**Published:** 2025-02-07

**Authors:** Gillian S Smith, Michael Fleming, Sally-Ann Cooper, Angela Henderson, Jill Pell, Craig Melville, Deborah Cairns

**Affiliations:** 1 School of Health and Wellbeing, University of Glasgow, Glasgow, UK

**Keywords:** Epilepsy, Asthma, Community child health, DIABETES & ENDOCRINOLOGY, Adolescents

## Abstract

**Abstract:**

**Objectives:**

To investigate hospitalisation rates for the ambulatory care-sensitive conditions of epilepsy, asthma and insulin-dependent diabetes in school-aged children and young people with intellectual disabilities in comparison with their peers.

**Design:**

Record-linkage cohort study. Scotland’s Pupil Census, 2008–2013, was used to identify pupils with and without intellectual disabilities and was linked with the Prescribing Information Service to identify pupils with epilepsy, asthma and insulin-dependent diabetes, and the Scottish Morbidity Records-01 to identify hospital admissions.

**Setting:**

The general child population of Scotland.

**Participants:**

School pupils aged 4–19 years; 18 278 with intellectual disabilities and 777 912 without intellectual disabilities.

**Outcomes:**

Overall, emergency and non-emergency hospitalisations for epilepsy, asthma and/or diabetes; and length of stay.

**Results:**

Epilepsy and asthma were more prevalent in pupils with intellectual disabilities (8.8% and 8.9%, respectively, compared with 0.8% and 6.9% among pupils without intellectual disabilities, p<0.001), whereas insulin-dependent diabetes was not (0.5% prevalence). After adjusting for prevalence, pupils with intellectual disabilities and epilepsy had more epilepsy-related admissions than their peers (adjusted Hazard Ratio (aHR) 2.24, 95% CI 1.97, 2.55). For emergency admissions, these stays were longer compared with controls (adjusted incidence rate ratio (aIRR) 2.77, 95% CI 2.13, 3.59). Pupils with intellectual disabilities and asthma had similar admission rates due to asthma as control pupils with asthma (aHR 0.81, 95% CI 0.62, 1.06), but emergency admissions were longer (aIRR 2.72, 95% CI 1.49, 4.96). Pupils with intellectual disabilities and insulin-dependent diabetes had similar admission rates to controls (aHR 0.94, 95% CI 0.63, 1.41) but with shorter admissions (aIRR 0.71, 95% CI 0.51, 0.99).

**Conclusions:**

Our findings suggest pupils with intellectual disabilities may receive poorer community healthcare than their peers for the common conditions of epilepsy and asthma. Hospital admissions are disruptive for both the child and their family. Epilepsy and asthma are associated with avoidable deaths; hence, a better understanding of these hospitalisations is important.

**STRENGTHS AND LIMITATIONS OF THIS STUDY**

- Large, national study.
- Identification of over 18 000 children and young people with intellectual disabilities.
- Diagnoses of epilepsy, asthma and insulin-dependent diabetes were based on dispensing of prescriptions.
- Cannot distinguish between mild and severe intellectual disabilities.
- Unable to investigate whether there are any ethnic variations.

## Introduction

 Ambulatory care-sensitive conditions are health conditions for which timely and effective community healthcare helps to reduce the risks of hospitalisation by preventing the onset of illness, controlling an acute episode of illness or managing an enduring condition.^1^ Examples include epilepsy, asthma and diabetes. General population studies have revealed an association between high rates of hospitalisations for ambulatory care-sensitive conditions and poor access to primary care.^2 3^ Intellectual disabilities are a group of conditions resulting in an IQ of less than 70, the need for daily support in adaptive functioning and onset in childhood. People with intellectual disabilities face barriers in accessing community health services.^4^,^6^ However, there has been little previous study of hospital admissions for ambulatory care-sensitive conditions in people with intellectual disabilities in comparison with the general population,^7^ particularly with regard to school-aged children and young people.

Previous studies have reported the frequency of all admissions (rather than specifically for ambulatory care-sensitive conditions) for preschool children with intellectual disabilities compared with the general population. One study, from the USA, reported on children with Down syndrome up to age 3 years, rather than all children and young people with intellectual disabilities,^8^ while another, from Western Australia, reported from birth to age 5 years.^9^ Both showed higher rates of hospitalisation than the control population, but they did not take into account different prevalences of conditions between the populations. Likewise, a large study using hospital billing data from four USA states investigated admissions of children aged under 18 years with a combined, heterogeneous range of intellectual and developmental disorders (eg, intellectual disabilities, autism, cerebral palsy).^10^ It found the relative risk of hospitalisation was 19.43 (18.56–20.34) for epilepsy and 3.60 (3.33–3.90) for asthma, but it did not take account of the different prevalences of these conditions. A further study focused specifically on ambulatory care-sensitive conditions.^11^ It included 8000 people of all ages (children and adults) with intellectual disabilities living in Manitoba, Canada, between 1999 and 2003. It reported hospitalisation rate ratios for 14 conditions combined of 6.38 (95% CI 5.30, 7.67) at ages 0–9 years, and 8.47 (95% CI 6.89, 10.42) at ages 10–19 years. For specifically asthma and diabetes, it adjusted for the prevalence of the condition, reporting rate ratios of 2.10 (95% CI 1.39, 3.16) for asthma and 3.73 (95% CI 2.63, 5.29) for diabetes; however, it did not report rates separately for children or young people for these conditions. A smaller study investigated 107 children and young people with ‘cognitive and developmental delays’ and 943 children and young people without, up to age of 18 years, in Quebec, Canada.^12^ It did not find any difference in hospitalisation ratios for ambulatory care-sensitive conditions between the two groups; however, the group with cognitive and developmental delays was very heterogeneous due to coding issues and included, for example, specific learning disabilities and speech and language disorders. A further study of 1148 children with intellectual disabilities and 2255 control children aged 2–24 years in South Carolina used hospital billing data to explore eight ambulatory care-sensitive conditions, but they did not include epilepsy.^13^ They reported more events in the children with intellectual disabilities: an incidence rate ratio (IRR) of 1.23 (1.05–1.44) for emergency room visits and an IRR of 2.62 (1.95–3.32) for inpatient admissions. Finally, a large observational study in England reported rates of emergency admissions for ambulatory care-sensitive conditions for people of all ages with intellectual disabilities, with some limited results for children and young people separately.^14^ Those aged 0–24 years in the study had 71.0 emergency admissions per 1000 person-years (95% CI 66.0, 76.4), compared with 15.2 per 1000 (95% CI 15.1, 15.4) in children/young people without intellectual disabilities. However, the study did not adjust for the prevalence of these conditions or provide further data, such as IRRs for this age group.^14^ Hence, the results of existing studies are not directly comparable, accounting for their contradictory findings: we have much to learn on this topic.

The aim of this study was to investigate hospitalisation rates for the ambulatory care-sensitive conditions of epilepsy, asthma and insulin-dependent diabetes in school-aged children and young people with intellectual disabilities, in comparison with similarly aged control children, taking account of the different prevalence of these conditions in the two groups. Epilepsy and asthma were selected as they occur commonly in children with intellectual disabilities. They are also long-term conditions; hence, the children establish a relationship with their primary healthcare professionals. The selected linked dataset that includes these conditions also holds data on insulin-dependent diabetes, hence its inclusion in our aim.

## Methods

### Data sources and record linkage

This study used administrative data from Scotland’s annual pupil census to identify all children and young people with intellectual disabilities in Scotland. The census is held by the Scottish Government and includes pupils from all local authority schools, including special schools and funded placements, covering an estimated 95% of schoolchildren across Scotland. The Pupil Census also contained records of any child receiving additional support needs at school due to intellectual disability. We used a strict definition of at least two records of support for intellectual disabilities (ie, 2 years) to identify children with intellectual disabilities. The entry date was defined as the date the second record of support for intellectual disabilities was accrued. The comparison group was formed of pupils with at least two census records, who did not have any records of intellectual disabilities or autism, with the date of their second census record assigned as the study entry date. Only pupils aged between 4 and 19 years at entry were included in the study. Information on sex and neighbourhood deprivation was also derived from the census; deprivation was ascertained using the Scottish Index of Multiple Deprivation (SIMD) 2012, which is based on individual postcode data. The SIMD score from the first census record was used for each pupil. Records were linked using probabilistic matching, based on sex, date of birth and postcode of residence, to administrative health datasets in Scotland, held by Public Health Scotland. The highest scoring match was used and unlikely or duplicate matches were excluded.

Information was extracted from the Prescribing Information System which recorded all prescriptions dispensed in Scotland between 1 January 2009 and 31 December 2013. Data were extracted on medications with British National Formulary (BNF) codes relevant to ambulatory care-sensitive conditions: epilepsy, asthma and insulin-dependent diabetes. Information was extracted from the Scottish Morbidity Records (SMR) dataset on acute inpatient and day case episodes (SMR-01), and maternity inpatient and day case episodes (SMR-02). These are episode-based datasets on all acute hospital admissions (SMR-01) and all maternity admissions (SMR-02) across Scotland, which record the admission and discharge dates; the main condition or diagnosis for the admission using International Classification of Diseases (ICD-10) codes; and whether the admission was an emergency or routine admission. Maternity records (SMR-02) were used to identify any child from a multiparous birth born in Scotland, who were then excluded to remove any potential mismatching between same-sex siblings with the same birthdate, as the linkage methodology could not distinguish between them.

### Exposure definitions

Dispensed prescriptions for disease-specific medications were used as a proxy measure for each condition, using methodology validated in previous studies.^15^,^18^ Pupils prescribed at least one antiepileptic drug (BNF section 4.8) were defined as having epilepsy. Pupils with more than one prescription during the same calendar year for an inhaled steroid and either a long-acting or short-acting β-agonist (BNF sections 3.1, 3.2 and 3.3) were defined as having asthma; pupils who only met one of these criteria were excluded. Pupils with at least one insulin prescription (BNF section 6.1.1) were defined as having insulin-dependent diabetes; pupils on oral antidiabetic medication only were excluded. All conditions were analysed exclusively. Pupils without recorded prescriptions for any of the other ambulatory care-sensitive conditions were assigned to the control group for the analysis. For the epilepsy, asthma and diabetes analyses, all pupils’ entry dates were re-assigned as the latest date out of either their index prescription or their index pupil census record.

### Outcome definitions

All prospective hospital admissions up to the censor date of 13 February 2015, or, if earlier, the date of death, or the date the pupil reached age 25 years, were extracted. Data were extracted for admissions due to epilepsy, status epilepticus or seizures (ICD-10 codes G40, G41 and R568); for asthma or status asthmaticus (ICD-10 codes J45 and J46); and for insulin-dependent diabetes (ICD-10 codes E10–E14). The length of stay in the hospital in days was calculated using the admission and discharge dates, with 1 day classified as a day-case admission to the hospital.

### Statistical analyses

We calculated the prevalence of three ambulatory care-sensitive conditions—epilepsy, asthma and diabetes—for pupils with and without intellectual disabilities. For each condition, group differences in sex and deprivation quintiles for those with and without intellectual disabilities were compared. The number of pupils admitted to hospital prospectively during the study and the mean number of admissions per person were compared for pupils with and without intellectual disabilities using χ^2^ tests and t-tests, respectively. For each of the ambulatory care-sensitive conditions, we reported the incidence rates (pupils with an incident hospitalisation, per 1000 pupils per year) for those with and without intellectual disabilities (including stratified for emergency and routine admissions). Univariate Cox proportional hazards models were used to assess the risk difference between pupils with, referent to without, intellectual disabilities. Cox models adjusted for age at study entry, sex and neighbourhood deprivation level were also employed. Median length of stay and proportion of day cases were compared for pupils with and without intellectual disabilities using Mann-Whitney U tests and χ^2^ tests, respectively. Zero-truncated negative binomial regression models were used to report differences in total length of stay for pupils with, referent to without, intellectual disabilities for admitted pupils only (minimum stay of 1 day) for each condition. Robust standard errors for IRR were used to adjust for multiple admissions per person. Statistical analyses were undertaken using Stata, V.15.0 (StataCorp).

### Patient and public involvement

This research was undertaken within the Scottish Learning Disabilities Observatory. Initially, a systematic review was completed on the topic.^7^ Its findings were presented to the steering committee of the Observatory, which included people with intellectual disabilities, representatives from two third-sector organisations for people with intellectual disabilities and family carers. The discussion that followed identified this area as one that should be taken forward for further research, and the study design was approved by the steering committee.

## Results

### Cohort demographics

18 278 (1.9%) pupils were recorded as having intellectual disabilities over the study period, 2009–2013, of whom 11 891 (65.1%) were male. The control group consisted of 777 912 pupils, of whom 389 160 (50.0%) were male. There were more pupils with intellectual disabilities living in areas of greater neighbourhood deprivation; 5822 (31.9%) in the most deprived quintile compared with 169 038 (21.7%) without intellectual disabilities. More detailed demographic information has previously been reported for this cohort.^19^

There were 3660 pupils who were in receipt of prescriptions for asthma but did not meet our full definition and so were excluded from the asthma analysis. 503 pupils were prescribed an oral antidiabetic drug but not insulin and were thus excluded from the diabetes analysis.

### Prevalence of conditions

Table 1 shows the prevalence of each condition in pupils with and without intellectual disabilities, using proxy definitions based on their prescribing information. Epilepsy (p<0.001) and asthma (p<0.001) had much higher prevalence among pupils with intellectual disabilities, whereas insulin-dependent diabetes (p=0.841) occurred at similar rates in both groups. 1608/18 278 (8.8%) pupils with intellectual disabilities had epilepsy and 6441/777 912 (0.8%) control pupils. 1621/18 196 (8.9%) pupils with intellectual disabilities had asthma and 53 363/774 334 (6.9%) control pupils. 94/18 238 (0.5%) pupils with intellectual disabilities had insulin-dependent diabetes and 3924/777 449 (0.5%) control pupils.

**Table 1:** Prevalence of epilepsy, asthma and diabetes for pupils with and without intellectual disabilities

Condition	Intellectual disabilities		Controls		P value	Total	
Epilepsy							
Prevalence	1608/18 278	8.8%	6441/7 77 912	0.8%	<0.001	8049/7 96 190	1.0%
Excluded pupils	0		0			0	
Asthma							
Prevalence	1621/18 196	8.9%	53 363/7 74 334	6.9%	<0.001	54 984/7 92 530	6.9%
Excluded pupils	82		3578			3660	
Insulin-dependent diabetes							
Prevalence	94/18 238	0.5%	3924/7 77 449	0.5%	=0.841	4018/7 95 687	0.5%
Excluded pupils	40		463			503	

### Hospital admissions

#### Epilepsy

Pupils with both intellectual disabilities and epilepsy had more frequent hospital admissions for all causes compared with control pupils with epilepsy (62% vs 37%, p<0.001). Detailed data on all-cause admissions can be found in the online supplemental table S1. Table 2 shows that pupils with intellectual disabilities and epilepsy had more admissions due to epilepsy compared with control pupils with epilepsy (adjusted Hazard Ratio (aHR) 2.24, 95% CI 1.97, 2.55). Although the overall duration of their admissions was longer, the difference was not statistically significant.

**Table 2:** Hospital admissions due to epilepsy, status epilepticus or seizures* among pupils with epilepsy, with and without intellectual disabilities, including IRRs, Cox proportional hazards models for risk of admission and zero-truncated negative binomial regression for total length of stay

All acute admissions due to epilepsy*	Intellectual disabilities and epilepsy†		Controls and epilepsy†		P value‡
Total pupils, n	1608		6441		
Total admissions, n	1134		1256		
Pupils admitted, n%	395	24.6%	581	9.0%	<0.001
Males admitted, n%	230	24.2%	285	10.9%	<0.001
Females admitted, n%	165	25.1%	296	7.7%	<0.001
Mean admissions per person (SD)	2.87	(4.7)	2.16	(2.5)	=0.003
N day cases, % admissions	401	35.4%	389	31.0%	=0.023
Length of stay, days, median (IQR)	2	(1, 3)	2	(1, 3)	<0.444
(excluding days cases)	3	(2, 4)	2	(2, 4)	<0.028
**Incidence of admission/1000personyears (95% CI)**	**Rate per1000(95%CI)**		**Rate per1000(95%CI)**		
All pupils	79.67	(72.19, 87.93)	32.40	(29.87, 35.14)	
Males	79.57	(69.92, 90.54)	36.91	(32.86, 41.45)	
Females	79.82	(68.53, 92.98)	28.99	(25.87, 32.49)	
**Cox PH models**	**HR (95% CI)**				
Intellectual disabilities	2.55	(2.25, 2.90)			
	**aHR** **§** **(95% CI)**				
Intellectual disabilities	2.24	(1.97, 2.55)			
**Length of stay models**	**IRR (95% CI)**				
All pupils admitted (n=2390)					
Intellectual disabilities	1.28	(0.84, 1.94)			
	**AdjustedIRR** **§** **(95% CI)**				
All pupils admitted (n=2390)					
Intellectual disabilities	1.32	(0.92, 1.89)			

t-test was used for mean admissions per person, Mann-Whitney U test was used for length of stay.

* ICD 10 codes G40, G41, R568.; a - adjusted for age at entry, () b –X test was used for comparing n pupils admitted, n day cases; t-test was used for mean admissions per person, Mann-Whitney U test was used for length of stay

† pPupils with anti-epileptic drug () prescription.

‡χ 2 test was used for comparing n pupils admitted, n day cases.

§ Adjusted for age at entry, sex and deprivation quintile Scottish Index of Multiple Deprivation.

aHRadjusted Hazard RatioICDInternational Classification of DiseasesIRRincidence rate ratio

For the pupils with intellectual disabilities and epilepsy, 864/1134 (76.2%) epilepsy admissions were emergency admissions, and 270/1134 (23.8%) were routine admissions. Among control pupils with epilepsy, 828/1256 (65.9%) epilepsy admissions were emergency admissions, and 428/1256 (34.1%) were routine admissions. There was an increased risk of epilepsy emergency admissions for pupils with intellectual disabilities compared with control pupils (aHR 2.50, 95% CI 2.15 to 2.91) and for routine epilepsy admissions (aHR 1.57, 95% CI 1.28 to 1.91). There was no significant interaction with sex (emergency admission; p_interaction_=0.112).

For emergency admissions, the pupils with intellectual disabilities and epilepsy had longer lengths of stay than the control pupils (IRR 2.77, 95% CI 2.13, 3.59). For routine admissions, the lengths of stay were not significantly different (IRR 0.74, 95% CI 0.53, 1.03).

#### Asthma

Pupils with both intellectual disabilities and asthma had more frequent all-cause hospital admissions than control pupils with asthma (33% vs 26%, p<0.001). Data on all-cause admissions can be seen in the online supplemental table S2. Table 3 shows that pupils with intellectual disabilities and asthma had fewer admissions due to asthma than did control pupils with asthma, but survival analysis showed no significant difference in the risk of admission (aHR 0.81, 95% CI 0.62, 1.06). Overall, their admissions were for a similar length of stay.

**Table 3:** Hospital admissions due to asthma or status asthmaticus* among pupils with asthma with and without intellectual disabilities, including incidence rates, Cox proportional hazards model for risk of admission and zero-truncated negative binomial regression for total length of stay

All admissions due to asthma*	Intellectual disabilities and asthma†		Controls and asthma†		P value‡
Total pupils, n	1621		53 363		
Total admissions, n	146		5340		
Pupils admitted, n%	56	3.5%	2661	4.9%	=0.005
Males admitted n%	42	3.7%	1453	4.9%	=0.085
Females admitted, n%	14	2.8%	1208	5.1%	=0.019
N day cases, % admissions	66	45%	1594	30%	<0.001
Length of stay, days, median (IQR)	2	1,3	2	1,3	=0.050
(Excluding days cases)	3	2,5	3	2,4	=0.009
**Incidence of admission/1000personyears (95% CI)**	**Rate per1000(95%CI)**		**Rate per1000(95%CI)**		
All pupils	10.76	(8.28, 13.98)	13.13	(12.64, 13.64)	
Male pupils	11.53	(8.52, 15.61)	12.62	(11.99, 13.29)	
Female pupils	8.95	(5.31, 15.11)	13.79	(13.04, 14.59)	
**Cox PH models**	**HR (95% CI)**				
Intellectual disabilities	0.77	(0.59, 1.00)			
	**aHR** **§** **(95% CI)**				
Intellectual disabilities	0.81	(0.62, 1.06)			
**Length of stay models**	**IRR (95% CI)**				
All pupils admitted (n=2717)					
Intellectual disabilities	1.88	(0.71, 4.92)			
	**AdjustedIRR** **§** **(95% CI)**				
All pupils admitted (n=2717)					
Intellectual disabilities	1.95	(0.78, 4.89)			

* ICD 10 codes J45, J46.

† Prescription for asthma (inhaled steroid and β-agonist).

‡ Xχ2 test was used for comparing n pupils admitted, n day cases; t-test was used for mean admissions per person, Mann-Whitney U test was used for length of stay.

§ aAdjusted for age at entry, sex and deprivation quintile ()Scottish Index of Multiple Deprivation.

aHRadjusted Hazard RatioICDInternational Classification of DiseasesIRRincidence rate ratio

For the pupils with intellectual disabilities and asthma, 95/146 (65.1%) asthma admissions were emergency admissions, and 51/146 (34.9%) were routine admissions. Among control pupils with asthma, 4889/5340 (91.6%) asthma admissions were emergency admissions, and 451/5340 (8.4%) were routine admissions. Among pupils with asthma, pupils with intellectual disabilities were at similar risk of emergency admissions (aHR 0.83, 95% CI 0.63, 1.08). Data for routine asthma admissions are not shown due to statistical disclosure, as the total 51 routine admissions were for a group of less than five pupils with intellectual disabilities; that is, almost all of the pupils with intellectual disabilities and asthma who were admitted had emergency admissions.

For emergency admissions, the pupils with intellectual disabilities and asthma had longer lengths of stay than control pupils (adjusted IRR (aIRR) 2.72, 95% CI 1.49, 4.96). Calculations were not undertaken for routine admissions.

#### Diabetes

Pupils with both intellectual disabilities and insulin-dependent diabetes had similar all-cause hospital admission rates compared with control pupils with insulin-dependent diabetes (56% admitted vs 52%, p<0.353). Data on all-cause admissions can be seen in the online supplemental table S3.

Table 4 shows that pupils with intellectual disabilities and insulin-dependent diabetes had fewer admissions due to diabetes than control pupils with insulin-dependent diabetes, but survival analysis shows no statistical difference (aHR 0.94, 95% CI 0.63 to 1.41). Overall, their admissions were of shorter length of stay (aIRR 0.71, 95% CI 0.51, 0.99).

**Table 4:** Hospital admissions due to diabetes* among pupils with insulin-dependent diabetes, with and without intellectual disabilities, including incidence rates, Cox proportional hazards models for the risk of admission and zero-truncated negative binomial regression for total length of stay

All admissions due to diabetes*	Intellectual disabilities and diabetes†		Controls and diabetes†		P value‡
Total pupils, n	94		3924		
Total admissions, n	54		3089		
Pupils admitted, n%	24	25.5%	1227	31.3%	=0.235
Males admitted, n%	15	27.3%	532	26.6%	0.908
Females admitted, n%	9	23.1%	695	36.2%	0.092
Mean admissions per person (SD)	2.25	(2.3)	2.52	(3.4)	=0.704
N day cases, % admissions	16	29.6%	545	17.6%	=0.023
Length of stay, days, median (IQR)	2	1,3	3	2,4	=0.031
(Excluding days cases)	3	2,4	3	2,4	=0.371
**Incidence of admission/1000personyears (95% CI)**	**Rate per1000(95%CI)**		**Rate per1000(95%CI)**		
All pupils	96.72	(64.83, 144.30)	95.76	(90.55, 10.27)	
Male pupils	102.88	(62.02, 170.65)	79.00	(72.57, 86.01)	
Female pupils	87.94	(45.76, 169.01)	114.33	(106.14, 123.16)	
**Cox PH models**	**HR (95% CI)**				
Intellectual disabilities	0.98	(0.65, 1.47)			
	**aHR** **§** **(95%CI)**				
	0.94	(0.63, 1.41)			
**Length of stay models**	**IRR (95% CI)**				
All pupils admitted, (n=1251)					
Intellectual disabilities	0.70	(0.51, 0.97)			
	**AdjustedIRR** **§** **(95%)**				
All pupils admitted (n=1251)					
Intellectual disabilities	0.71	(0.51, 0.99)			

* ICD 10 codes E10-–E14.

† Prescription for insulin.

‡χ X2 test was used for comparing n pupils admitted, n day cases; t-test was used for mean admissions per person, Mann-Whitney U test was used for length of stay.

§ aAdjusted for age at entry, sex and deprivation quintile ()Scottish Index of Multiple Deprivation.

aHRadjusted Hazard RatioICDInternational Classification of DiseasesIRRincidence rate ratio

For the pupils with intellectual disabilities and insulin-dependent diabetes, 47/54 (87.0%) diabetes admissions were emergency admissions, and 7/54 (13.0%) were routine admissions. Among control pupils with insulin-dependent diabetes, 2849/3089 (92.2%) diabetes admissions were emergency admissions, and 240/3089 (7.8%) were routine admissions. Among pupils with insulin-dependent diabetes, pupils with intellectual disabilities were at similar risk of emergency admissions (aHR 0.83, 95% CI 0.54, 1.30) and of routine admissions (aHR 1.85, 95% CI 0.87, 3.94).

For emergency admissions, pupils with intellectual disabilities had shorter lengths of stay than the control pupils (aIRR 0.67, 95% CI 0.47, 0.95). Calculations were not undertaken for routine admissions.

## Discussion

### Principal findings and interpretation

For two of the three ambulatory care-sensitive conditions we investigated (epilepsy and asthma), our findings suggest that pupils with intellectual disabilities receive poorer community healthcare than control pupils. Among pupils with epilepsy, those who also have intellectual disabilities are at higher risk of both emergency and routine hospital admissions for epilepsy than control pupils and spend longer in hospital following emergency admissions. Among pupils with asthma, those who also had intellectual disabilities spent longer in hospital following emergency admissions. In contrast, they spent less time in hospital following emergency admissions for diabetes. We consider our findings novel, as there is little previous research with which to compare them.

There are several potential interpretations of these findings. The higher risk of epilepsy admissions in pupils with intellectual disabilities could reflect them having more severe epilepsy than control pupils, poorer management of their epilepsy in the community or both. The longer duration of emergency asthma admissions for pupils with intellectual disabilities suggests that their asthma may be more difficult to resolve once they are admitted. This could plausibly be due to delayed admission, with poorer community management being tolerated for longer than in control pupils.

While the shorter duration of admissions for diabetes in pupils with intellectual disabilities might be explained by better management in the community, it is more likely to be explained by the fact that pupils with intellectual disabilities are less likely to self-administer their insulin than control pupils. Given that adherence is lower among young people,^20^ the administration of medication by parents or carers may improve day-to-day management in the community and/or mean that changes to management in hospital are quicker to implement.

### Comparison with the existing literature

Epilepsy and asthma have previously been reported to be more common among children and young people with intellectual disabilities. We found similar rates of insulin-dependent diabetes in the two groups.

It is difficult to draw comparisons of hospitalisation ratios with previous literature due to study design differences. One study compared child/young person hospitalisation data on 14 ambulatory care-sensitive conditions combined, showing them to be more common in those with intellectual disabilities.^11^ While they were able to account for differences in population prevalence rates for asthma and diabetes in their further calculations, they did not report the ratios separately for children and young people. Some studies we referenced had populations that are not directly comparable to the children and adolescents with intellectual disabilities in our study.^10 12^ Some studies did not adjust for the different prevalence rates,^8^,^10^ and some studied younger, preschool children only, so are not comparable to our study.^8 9^

### Strengths and limitations

A strength of the study is its large size, covering all of Scotland, with over 18 000 children and young people with intellectual disabilities. The diagnoses of epilepsy, asthma and insulin-dependent diabetes were based on the dispensing of prescriptions; these conditions require drug treatment, as they are otherwise life-threatening, so this method of identification should be reasonably robust. We used school records to identify the children and young people with intellectual disabilities and therefore cannot distinguish between mild and severe intellectual disabilities. We were unable to investigate whether there were any ethnic variations.

### Implications

Our findings suggest that pupils with intellectual disabilities may receive poorer community healthcare than their peers for epilepsy and asthma. These are common conditions in children and young people with intellectual disabilities. It has previously been reported, almost exclusively through qualitative research, that adults with intellectual disabilities receive poorer community healthcare, with many issues contributing to this, including the sharing of information within and between care teams. For most children and young people, their healthcare is supported by their parents rather than care teams, so the disparity in the quality of epilepsy and asthma care is important to note and understand. Poor inhaler technique may be an issue for some children with intellectual disabilities but is not insurmountable as AeroChambers, and the larger nebuhalers and Volumatic Spacer devices are available to aid coordination once the issue has been identified. Additionally, electric or gas-driven nebulisers can be used for bigger doses and to deliver the medication deeper into the chest.

People with ambulatory care-sensitive conditions ideally should not be admitted to hospital. If admitted, they may experience further barriers to care, including those related to staff knowledge, skills and attitudes,^21^ highlighting the need for support for secondary care staff.

Hospital admissions are disruptive to child development and education, and stressful for both the child and their family. In addition, epilepsy and asthma are associated with avoidable deaths, hence a better understanding of hospitalisation for these ambulatory care-sensitive conditions is particularly important. Parents and teachers of children and young people with these conditions may benefit from greater support and information.

## supplementary material

10.1136/bmjopen-2024-088809 online supplemental file 1

## Data Availability

Data may be obtained from a third party and are not publicly available.
