# Supplementary material for: Hospitalisation rates for epilepsy, asthma and insulin-dependent diabetes in 796 190 school-aged children and young people with and without intellectual disabilities: a record-linkage cohort study
Source: BMJ Open. 2025 Feb 7;15(2):e088809. doi: 10.1136/bmjopen-2024-088809 (PMC11843016; doi:10.1136/bmjopen-2024-088809)
Supplement: online supplemental file 1 [file bmjopen-15-2-s001.docx]

# Supplementary tables:

Table S1. All-cause admissions among pupils with epilepsy with, and without, intellectual disabilities, including incidence rates, Cox proportional hazards models for risk of admission for pupils with epilepsy with, versus without, intellectual disabilities

| **All-cause admissions** | **Pupils with intellectual disabilities and epilepsy** | | | **Controls with epilepsy** | | | **p-value^b^** |
| --- | --- | --- | --- | --- | --- | --- | --- |
| Total pupils, n | 1,608 |  |  | 6,441 |  |  |  |
| Total admissions, n | 6,235 |  |  | 7,678 |  |  |  |
| Pupils admitted, n % | 985 | 61.3% |  | 2,366 | 36.7% |  | p<0.001 |
| Mean admissions per person, (sd) | 6.33 | (15.9) |  | 3.25 | (5.6) |  | p<0.001 |
| Incident admissions / 1,000 (95% CI) – All pupils | 316.06 | (296.93, 336.43) | | 171.97 | (165.18, 179.04) | |  |
| - Males | 301.28 | (277.50, 327.11) | | 155.32 | (145.69, 165.58) | |  |
| - Females | 338.70 | (307.70, 372.82) | | 185.01 | (175.65, 194.85) | |  |
| **All emergency admissions** |  |  |  |  |  |  |  |
| Total admissions, n | 2,723 |  |  | 3,855 |  |  |  |
| Total pupils admitted, n % | 686 | 42.6% |  | 1,538 | 23.9% |  | p<0.001 |
| Mean admissions per person, (sd) | 3.97 | (5.6) |  | 2.51 | (3.7) |  | p<0.001 |
| Incident admissions / 1,000 (95% CI) – All pupils | 164.71 | (152.83, 177.51) | | 96.42 | (91.72, 101.36) | |  |
| - Males | 160.56 | (145.56, 177.12) | | 86.49 | (79.87, 93.65) | |  |
| - Females | 170.84 | (152.18, 191.80) | | 104.20 | (97.72, 111.11) | | |
| **All routine admissions** |  |  |  |  |  |  |  |
| Total admissions, n | 3,512 |  |  | 3,823 |  |  |  |
| Total pupils admitted, n % | 733 | 45.6% |  | 1490 | 23.1% |  |  |
| Mean admissions per person, (sd) | 4.79 | (17.0) |  | 2.57 | (4.5) |  | p<0.001 |
| Incident admissions / 1,000 (95% CI) – All pupils | 188.02 | (174.89, 202.13) | | 93.52 | (88.89, 98.39) | |  |
| - Males | 178.24 | (161.91, 196.22) | | 85.41 | (78.80, 92.57) | |  |
| - Females | 202.60 | (181.48, 226.18) | | 99.76 | (93.45, 106.50) | |  |
| **PH Cox models All-cause admissions:** | **HR (95% CI)** | | |  | | |  |
| **All admissions** – Intellectual disabilities | 1.82 | (1.69, 1.96) | |  |  | |  |
| **Emergency admissions** – Intellectual disabilities | 1.74 | (1.59, 1.91) | |  |  | |  |
| **Routine admissions** – Intellectual disabilities | 2.04 | (1.86, 2.22) | |  |  | |  |
|  | **aHR^a^ (95%CI)** | | |  |  | |  |
| **All admissions** – Intellectual disabilities | 1.82 | (1.69, 1.96) | |  |  | |  |
| **Emergency admissions** – Intellectual disabilities | 1.75 | (1.60, 1.92) | |  |  | |  |
| **Routine admissions** – Intellectual disabilities | 2.01 | (1.83, 2.20) | |  |  | |  |

a – adjusted for age, sex, age at entry, and SIMD b – X^2^ test was used for comparing n pupils admitted; t-test was used for mean admissions per person

Table S2. All-cause admissions among pupils with asthma with, and without, intellectual disabilities, including incidence rates, Cox proportional hazards models for risk of admission for pupils with asthma with versus without intellectual disabilities

| **All-cause admissions:** | **Pupils with intellectual disabilities and asthma** | | | **Controls with asthma** | | | **p-value^b^** |
| --- | --- | --- | --- | --- | --- | --- | --- |
| Total pupils, n | 1,621 |  |  | 53,363 |  |  |  |
| Total admissions, n | 2067 |  |  | 27,910 |  |  |  |
| Total pupils admitted, n % | 533 | 32.9% |  | 13,737 | 25.7% |  | p<0.001 |
| Mean admissions per person, (sd) | 3.88 | (11.0) |  | 2.03 | (3.5) |  | p<0.001 |
| Incident admissions / 1,000 (95% CI)  – All pupils | 132.03 | (121.28, 143.73) | | 78.87 | (77.56, 80.20) | |  |
| - Males | 121.38 | (109.30, 134.78) | | 75.38 | (73.69, 77.11) | |  |
| - Females | 158.66 | (137.26, 183.39) | | 83.50 | (81.46, 85.60) | |  |
| **All emergency admissions** |  |  |  |  |  |  |  |
| Total admissions, n | 977 |  |  | 16,794 |  |  |  |
| Total pupils admitted, n % | 348 |  |  | 9,290 |  |  | p<0.001 |
| Mean admissions per person, (sd) | 2.81 | (4.3) |  | 1.81 | (2.7) |  | p<0.001 |
| Incident admissions / 1,000 (95% CI)  – All pupils | 77.03 | (69.35, 85.56) | | 49.86 | (48.86, 50.89) | |  |
| - Males | 71.90 | (63.20, 81.79) | | 48.04 | (46.73, 49.37) | |  |
| - Females | 89.66 | (74.80, 107.47) | | 52.27 | (50.71, 53.87) | |  |
| **All routine admissions** |  |  |  |  |  |  |  |
| Total admissions, n | 1,090 |  |  | 11,116 |  |  |  |
| Total pupils admitted, n % | 331 | 20.4 |  | 6,742 | 12.6% |  | p<0.001 |
| Mean admissions per person, (sd) | 3.29 | (12.4) |  | 1.65 | (2.3) |  | p<0.001 |
| Incident admissions / 1,000 (95% CI)  – All pupils | 73.15 | (65.68, 81.48) | | 34.88 | (34.06, 35.73) | |  |
| - Males | 65.82 | (57.51, 75.32) | | 32.14 | (31.10, 33.22) | |  |
| - Females | 91.00 | (76.09, 108.82) | | 38.50 | (37.19, 39.86) | |  |
| **PH Cox Models All-cause admissions:** | **HR** **(95% CI)** | | |  | | | |
| **All admissions** – Intellectual disabilities | 1.59 | (1.45, 1.73) | |  |  |  |  |
| **Emergency admissions** – Intellectual disabilities | 1.48 | (1.33, 1.64) | |  |  |  |  |
| **Routine admissions** – Intellectual disabilities | 2.01 | (1.80, 2.24) | |  |  |  |  |
|  | **aHR**^a^ **(95% CI)** | | |  |  |  |  |
| **All admissions** – Intellectual disabilities | 1.56 | (1.43, 1.71) | |  |  |  |  |
| **Emergency admissions** – Intellectual disabilities | 1.45 | (1.30, 1.61) | |  |  |  |  |
| **Routine admissions** – Intellectual disabilities | 2.00 | (1.79, 2.23) | |  |  |  |  |

a – adjusted for age, sex, age at entry, and SIMD b – X^2^ test was used for comparing n pupils admitted, n day cases; t-test was used for mean admissions per person, Mann-Whitney U test was used for length of stay

Table S3 . All-cause admissions among pupils with insulin-dependent diabetes with, and without, intellectual disabilities, including incidence rates, Cox proportional hazards models for risk of admission for pupils with diabetes, with versus without intellectual disabilities

| **All-cause admissions:** | **Intellectual Disabilities & diabetes** | | | **Controls with diabetes** | | | **p-value^b^** |
| --- | --- | --- | --- | --- | --- | --- | --- |
| Total pupils, n | 94 |  |  | 3,924 |  |  |  |
| Total admissions, n | 245 |  |  | 6,729 |  |  |  |
| Total pupils admitted, n % | 53 | 56.4% |  | 2,022 | 51.5% |  | p=0.353 |
| Mean admissions per person, (sd) | 4.62 | (9.5) |  | 3.33 | (5.8) |  | p=0.115 |
| Incident admissions / 1,000 (95% CI)  – All pupils | 314.01 | (239.89, 411.02) | | 197.16 | (188.75, 205.94) | |  |
| - Males | 342.04 | (244.40, 478.69) | | 174.45 | (163.68, 185.92) | |  |
| - Females | 273.84 | (174.67, 429.32) | | 222.70 | (209.78, 236.42) | |  |
| **All emergency admissions** |  |  |  |  |  |  |  |
| Total admissions, n | 100 |  |  | 5,222 |  |  |  |
| Total pupils admitted, n % | 34 | 36.2% |  | 1,761 | 44.9% |  | p=0.093 |
| Mean admissions per person, (sd) | 2.94 | (2.6) |  | 2.97 | (3.7) |  | p=0.364 |
| Incident admissions / 1,000 (95% CI) – All pupils | 151.71 | (108.40, 212.32) | | 158.20 | (150.98, 165.76) | |  |
| **All routine admissions** |  |  |  |  |  |  |  |
| Total admissions, n | 145 |  |  | 1,507 |  |  |  |
| Total pupils admitted, n % | 34 | 36.2% |  | 750 | 19.1% |  | p<0.001 |
| Mean admissions per person, (sd) | 4.26 | (10.8) |  | 2.01 | (6.1) |  | p=0.045 |
| Incident admissions / 1,000 (95% CI) – All pupils | 22.85 | (10.90, 47.94) | | 13.36 | (11.67, 15.30) | |  |
| **PH Cox Models All-cause admissions:** | **HR** **(95% CI)** | | |  | | | |
| **All admissions** – Intellectual disabilities | 1.46 | (1.11, 1.92) | |  |  |  |  |
| **Emergency admissions** – Intellectual disabilities | 0.91 | (0.65, 1.28) | |  |  |  |  |
| **Routine admissions** – Intellectual disabilities | 2.61 | (1.85, 3.69) | |  |  |  |  |
|  | **aHR**^a^ **(95% CI)** | | |  |  |  |  |
| **All admissions** – Intellectual disabilities | 1.44 | (1.10, 1.90) | |  |  |  |  |
| **Emergency admissions** – Intellectual disabilities | 0.90 | (0.64, 1.26) | |  |  |  |  |
| **Routine admissions** – Intellectual disabilities | 2.61 | (1.85, 3.69) | |  |  |  |  |

a – adjusted for age, sex, age at entry, and SIMD b – X^2^ test was used for comparing n pupils admitted, n day cases; t-test was used for mean admissions per person
