# Supplementary material for: Hospitalisation rates for epilepsy, asthma and insulin-dependent diabetes in 796 190 school-aged children and young people with and without intellectual disabilities: a record-linkage cohort study
Source: BMJ Open. 2025 Feb 7;15(2):e088809. doi: 10.1136/bmjopen-2024-088809 (PMC11843016; doi:10.1136/bmjopen-2024-088809)
Supplement: Supplementary file 2 [file bmjopen-2024-088809.reviewer_comments.pdf]

PEER REVIEW HISTORY

BMJ Open publishes all reviews undertaken for accepted manuscripts. Reviewers are asked to complete a checklist review form and are provided with free text boxes to elaborate on their assessment. These free text comments are reproduced below.

ARTICLE DETAILS

Title (Provisional)

Hospitalisation rates for epilepsy, asthma, and insulin-dependent diabetes in 796,190 school aged children and young people with, and without, intellectual disabilities: a record-linkage cohort study

Authors

Smith, Gillian S; Fleming, Michael; Cooper, Sally-Ann; Henderson, Angela; Pell, Jill; Melville, Craig; Cairns, Deborah

---

VERSION 1 - REVIEW

Reviewer **1**

Name **Meid, Andreas**

Affiliation **Heidelberg University Hospital, Dept. of Clinical Pharmacology & Pharmacoepidemiology**

Date **28-Jun-2024**

COI **None declared**

Short summary of the study

The aim of the study was to determine the hospitalisation rate for epilepsy, asthma and insulin-dependent diabetes among pupils aged 4-19 years with and without intellectual disabilities. The authors used a record linkage cohort approach involving several data sources:

(1) Scotland's annual student census (2008-2013) to identify pupils with intellectual disabilities.

(2) The Prescribing Information System (PIS). BNF codes were used as a proxy for diagnosis.

(3) Scottish Morbidity Records (SMR), specifically SMR-01 and SMR-02. Data were extracted using ICD10 codes.

Epilepsy and asthma were more prevalent conditions among pupils with intellectual disabilities. Epilepsy was the only condition with an aHR indicating an increased (risk) admission. Adjusted IRR indicating that pupils with intellectual disabilities and epilepsy/asthma had emergency admissions rates of longer duration. Overall, the authors,

based on their findings, claim that pupils with intellectual disabilities have poorer community health. They also point out that there are no previous studies to compare their findings, which makes the study novel.

General comment

The study is valuable due to its large and representative Scottish cohort, bridging knowledge gaps on this specific long-term condition and age group studied. The paper is clear and well-structured, but some issues must be addressed before publication.

Methods

• The outcome definition is inserted in the statistical method. It would benefit the reader to add an outcome definition similar to what was done with the exposure definitions and the timeframe.

• Page 7 of 29, Exposure definition. It's mentioned that "encashed prescription" (claim prescription?) was used as a proxy. Then, the term "prescription" also was used as a synonym. Being unfamiliar with the Scottish health system, is an encashed prescription = a prescription? This is relevant because it raises the question: Are all prescribed patients claiming their prescriptions? Reference No. 15 also used "dispensed prescriptions" instead of encashed, which is a more standard name in EHR.

• On page 8 of 29, lines 12-14, it's mentioned that pupils with no sensitive long-term conditions were assigned to the control group for the analysis. Could you please explain that further?

• Page 8 of 29, lines 14-17, mentions the pupil's entry dates. Is this the entry date into the cohort? If yes, the word "diagnosis" is used when, in reality, the proxies did not aim to identify the diagnosis date but determine whether the pupil has the disease at certain period. Instead, it should point out a date related to the prescription.

Results

• The hospital admissions section used the phrases: "…pupils with intellectual disabilities and epilepsy had more admissions and day case admissions…" and "…pupils with intellectual disabilities and asthma/diabetes had fewer admissions and day case admissions". This should be more informative since this might lead to misinterpretation. Admission results are more straightforward when checking the tables, but it does not occur when checking the “N day cases” items.

Acknowledgements: Ignacio Leiva-Escobar helped me to complete this review.

**Reviewer**	**2**
**Name**	**Cramer, Joyce A.**

Affiliation Yale University School of Medicine, Psychiatry
Date 24-Sep-2024
COI [None indicated].

The aim of this study was to investigate hospitalization rates for the ambulatory care in sensitive conditions of epilepsy, asthma, and insulin-dependent diabetes in school age children and young people with intellectual disabilities.

Differences in admission rates for epilepsy may be attributed to school staff fear of seizures. Most children can be stabilized quickly post-seizure, with rapid discharge. In contrast, asthma patients may need more medical adjustment. It would have been interesting to know whether epilepsy patients took medication themselves. Nonetheless, it is not uncommon for parents to forget to give meds to children. Treatment for acute asthma episodes could be administered by the school nurse who could judge that the child is well enough to go home instead of to hospital. As noted, insulin treatment would be carefully monitored and less likely to result in hypo- or hyper-glycemia requiring medical attention. Inclusion of diabetes in analyses is interesting but is not parallel to epilepsy and asthma 'attacks' that commonly frighten onlookers.

VERSION 1 - AUTHOR RESPONSE

Reviewer: 1
Dr. Andreas Meid, Heidelberg University Hospital
Comments to the Author:
Short summary of the study

The aim of the study was to determine the hospitalisation rate for epilepsy, asthma and insulin-dependent diabetes among pupils aged 4-19 years with and without intellectual disabilities. The authors used a record linkage cohort approach involving several data sources:
(1) Scotland's annual student census (2008-2013) to identify pupils with intellectual disabilities.
(2) The Prescribing Information System (PIS). BNF codes were used as a proxy for diagnosis.
(3) Scottish Morbidity Records (SMR), specifically SMR-01 and SMR-02. Data were extracted using ICD10 codes.

Epilepsy and asthma were more prevalent conditions among pupils with intellectual disabilities. Epilepsy was the only condition with an aHR indicating an increased (risk) admission. Adjusted IRR indicating that pupils with intellectual disabilities and epilepsy/asthma had emergency admissions rates of longer duration. Overall, the authors, based on their findings, claim that pupils with intellectual disabilities have poorer community health. They also point out that there are no previous studies to compare their findings, which makes the study novel.

General comment

The study is valuable due to its large and representative Scottish cohort, bridging knowledge gaps on this specific long-term condition and age group studied. The paper is clear and well-structured, but some issues must be addressed before publication.

Methods

• The outcome definition is inserted in the statistical method. It would benefit the reader to add an outcome definition similar to what was done with the exposure definitions and the timeframe.

Thank you for your comments. An outcome definitions has now been included in the methods section.

• Page 7 of 29, Exposure definition. It's mentioned that "encashed prescription" (claim prescription?) was used as a proxy. Then, the term "prescription" also was used as a synonym. Being unfamiliar with the Scottish health system, is an encashed prescription = a prescription? This is relevant because it raises the question: Are all prescribed patients claiming their prescriptions? Reference No. 15 also used "dispensed prescriptions" instead of encashed, which is a more standard name in EHR.

Thank you, for your comment - this has now been amended from encashed to dispensed for clarity.

• On page 8 of 29, lines 12-14, it's mentioned that pupils with no sensitive long-term conditions were assigned to the control group for the analysis. Could you please explain that further?

Thank you, this was an error -and has been amended to the following:*"Pupils without recorded prescriptions for any other ambulatory care-sensitive conditions were assigned to the control group for the analysis"* This refers to the exclusion of pupils meeting criteria of multiple exposure definitions (e.g pupils with both epilepsy and asthma)

• Page 8 of 29, lines 14-17, mentions the pupil's entry dates. Is this the entry date into the cohort? If yes, the word "diagnosis" is used when, in reality, the proxies did not aim to identify the diagnosis date but determine whether the pupil has the disease at certain period. Instead, it should point out a date related to the prescription.

Thank you, we recognise this wording should be improved for clarity and accuracy -the term diagnosis has been removed and replaced with index prescription.

Results

• The hospital admissions section used the phrases: "...pupils with intellectual disabilities and epilepsy had more admissions and day case admissions..." and "...pupils with intellectual disabilities and asthma/diabetes had fewer admissions and day case admissions". This should be more informative since this might lead to misinterpretation. Admission results are more straightforward when checking the tables, but it does not occur when checking the "N day cases" items.

Thank you, this has now been clarified to include just the term "admissions" rather than admissions and day case admissions" – since the results refers to any admission included in the study. This added detail was originally included to emphasise the inclusion of both inpatient and single day cases in the outcome definition.

Acknowledgements: Ignacio Leiva-Escobar helped me to complete this review.

Reviewer: 2
Dr. Joyce A. Cramer, Yale University School of Medicine
Comments to the Author:
The aim of this study was to investigate hospitalization rates for the ambulatory care in sensitive conditions of epilepsy, asthma, and insulin-dependent diabetes in school age children and young people with intellectual disabilities.

Differences in admission rates for epilepsy may be attributed to school staff fear of seizures. Most children can be stabilized quickly post-seizure, with rapid discharge. In contrast, asthma patients may need more medical adjustment. It would have been interesting to know whether epilepsy patients took medication themselves. Nonetheless, it is not uncommon for parents to forget to give meds to children. Treatment for acute asthma episodes could be administered by the school nurse who could judge that the child is well enough to go home instead of to hospital. As noted, insulin treatment would be carefully monitored and less likely to result in hypo- or hyper-glycemia requiring medical attention. Inclusion of diabetes in analyses is interesting but is not parallel to epilepsy and asthma 'attacks' that commonly frighten onlookers.

** ** **

Thank you for your comments.

COI statements:

Reviewer: 1
Competing interests: None declared.

Reviewer: 2
Competing interests: [None indicated].

VERSION 2 - REVIEW

Reviewer 1
Name Meid, Andreas
Affiliation Heidelberg University Hospital, Dept. of Clinical Pharmacology & Pharmacoepidemiology
Date 20-Nov-2024
COI

Thank you for considering the comments. No further comments
